# Comparison of methods to estimate ruminal degradation and intestinal digestibility of protein in hydrolyzed feather meal with or without blood

**DOI:** 10.3168/jdsc.2021-0139

**Published:** 2022-02-10

**Authors:** K.K. Buse, D.L. Morris, H.L. Diaz, O.R. Drehmel, P.J. Kononoff

**Affiliations:** 1 Department of Animal Science, University of Nebraska-Lincoln, Lincoln 68503; 2 Milk Specialties Global, Eden Prairie, MN 55344

## Abstract

Hydrolyzed feather meal (HFM) is a feedstuff high in rumen undegraded protein (RUP) that can be used as an effective source of metabolizable protein for dairy cattle. Because the production process may vary, the rumen degradability and intestinal digestibility of HFM may also vary. Additionally, some processes may incorporate additional blood into the final product to result in feather meal with poultry blood. To determine the rumen degradability and intestinal digestibility of these products, several laboratory assays can be used; the common assays are the mobile bag (MOB), modified three-step (MTS), and Ross (ROS) assays. Although all 3 assays determine RUP digestibility, they vary in whether they are performed in situ, in vitro, or both. The objective of this study was to evaluate the ruminal degradability and intestinal digestibility of HFM originating from processes that differ in their inclusion of blood, and to compare the MOB, MTS, and ROS assays. Ten samples of HFM, which were identified by the suppliers as HFM with little blood (n = 5) and with more blood (n = 5), were spot-sampled, collected from 10 production plants across the United States, and subjected to all 3 assays. Assay type had an effect on RUP, total-tract crude protein (CP) digestibility, and the amount of RUP digested. A significant effect was observed on RDP and RUP concentrations for blood inclusion; no effect was detected for total-tract CP digestibility. We found no difference in RUP digestibility for assay or blood inclusion. There was also no interaction of the effect of assay or blood inclusion. Results suggest that even though there are differences in chemical composition in HFM associated with the inclusion of blood, such as ash and crude fat, few if any differences are observed in intestinal digestion of protein. Although the assays varied in their estimates of rumen undegraded protein, MOB and MTS yielded the most similar values. However, all 3 assays resulted in similar estimates of RUP digestibility.

## Highlights

• The mobile bag, modified three-step, and Ross assays estimate RUP digestibility.
• The 3 assays were compared using feather meal with lower and higher contents of blood.
• Assay had no effect on RUP digestibility.
• Differences in estimated RUP led to differences in the amount of digested RUP.
• Feather meal with more blood had higher RUP digestibility.

## Graphical Abstract

**Figure:**
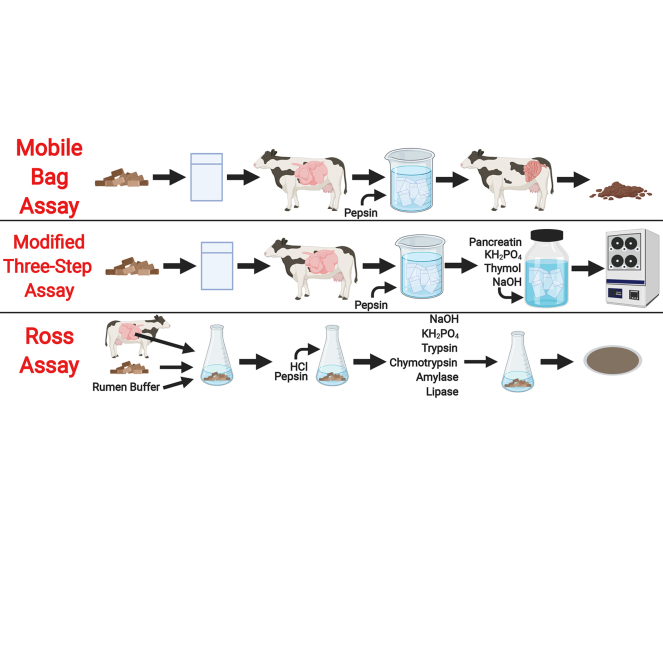
**Summary:** Using 3 different assays, this study evaluated the degradability and digestibility of protein in hydrolyzed feather meal. Traditionally, when hydrolyzed feather meal was produced, coagulated poultry blood was added before the drying phase; however, now some renderers separate the feathers and blood and sell them separately. These 2 types of commercially available hydrolyzed feather meal were evaluated. Although the assays differed in the estimates of rumen degradability of protein, intestinal digestibility of rumen undegraded protein (RUP) estimates were not different. We observed little difference in digestibility with the inclusion of blood, but rumen degradability was greater for feather meal not containing blood. No difference was observed in the digestibility of RUP between feather meal with and without blood. These results suggest that estimation of the intestinal digestibility of RUP is similar across assays and the inclusion of blood does not alter digestibility. However, due to differences in rumen degradability, the amount of RUP digested was significantly different across assays.

Feeding byproducts to ruminants has been practiced for centuries (Grasser et al., 1995). Not only are byproducts usually a cost-effective source of nutrients (Bradford and Mullins, 2012), they also contribute toward a sustainable industry by using nutrients that would otherwise be disposed of (Iriondo-DeHond et al., 2018). Hydrolyzed feather meal (**HFM**) is a byproduct of the rendering industry and has a high CP content on a DM basis (~85%), and approximately 65% of this protein is RUP, with an intestinal digestibility of 65% (NRC, 2001). In some cases, increasing the inclusion of HFM in rations led to a decrease in milk protein and DMI (Harris et al., 1992; Moss et al., 1995; Stahel et al., 2014; Morris et al., 2020). These results suggest that a lack of availability of protein and specific characteristics in the AA profile could explain in part the reduction in milk protein. Processing and handling of blood, such as the drying method used, as well as variations in the hydrolysis process of the feathers, such as length of process and whether blood was incorporated before hydrolysis, may affect digestibility (Meeker and Hamilton, 2006).

Traditionally, coagulated poultry blood was added back to the HFM before the drying phase used to produce HFM. However, because the market value of blood is greater than that of HFM itself, some renderers have begun to keep these components separate for individual sale. On a DM basis, blood meal has CP and RUP contents that are generally greater than those of HFM (95% and 77%, 4.0% of BW, respectively; NRC, 2001). This is not always true because the RUP content of blood meal has been shown to range from 14% to 70% (Paz et al., 2014). However, the NRC (2001) reports that HFM with some viscera has a RUP content of 65% CP and a Lys content of 2.90% of CP. Blood meal, depending on the drying method, can also have a higher RUP digestibility (**dRUP**) than HFM (80%, ring dried; 65%, batch dried; 65%, HFM; NRC, 2001). Lysine is a limiting EAA in many rations fed to lactating dairy cattle (Schwab et al., 1992), and blood meal is a significant source of metabolizable Lys that is lacking in HFM (NRC, 2001). There are limited data on how poultry blood meal compares with beef or porcine blood meal in terms of digestibility and AA content.

A challenge with feeding byproducts is that chemical composition and nutrient digestibility can vary depending upon the ingredient (Ertl et al., 2015) and the manufacturing process (Eggum, 1970; Kramer et al., 1978; Liu, 2011). This leads to challenges in ration formulation and can affect feed value. For HFM, coagulated blood can either be removed or allowed to remain with the product during the rendering process (Meeker and Hamilton, 2006). The variation in degradation and digestibility of some byproducts may require regular feed testing rather than users relying on values provided in feed libraries. To estimate protein and AA digestibility, Hvelplund (1985) developed the mobile bag assay (**MOB**), which is conducted almost entirely in situ with rumen incubation and passage of porous bags containing feedstuffs through the intestines after being inserted into the duodenum through a cannula. To minimize labor and cost as well as to minimize animal experimental use, Calsamiglia and Stern (1995) developed a 3-step procedure that still included in situ rumen incubation and used a pepsin and hydrochloric acid bath to mimic abomasal digestion. However, intestinal digestibility was determined in vitro with a pancreatin solution (a mixture of pancreatic enzymes) in centrifuge tubes. The assay was later modified by Gargallo et al. (2006) to use a Daisy^II^ incubator (Ankom Technologies) and a buffer-enzyme solution containing pancreatin and thymol, which acts to suppress microbial activity. This modified assay is commonly referred to as the modified three-step (**MTS**) assay. A concern with both the MOB and MTS assays is the use of nylon bags to contain the feeds throughout the entire assay. Not only can the bags potentially create a barrier to rumen microbes but loss of sample due to washout is a concern and affects estimated digestibility (Ross, 2013). There is also concern for bacterial contamination, which alters the estimates of rumen degradability and intestinal digestibility (Mathers and Aitchison, 1981; Beckers et al., 1995). More recently, Ross et al. (2013) developed an assay that can be performed entirely in vitro to isolate the RUP residue and then estimate the intestinal digestibility of this residue. This assay is commonly referred to as the Ross assay (**ROS**). Unlike MOB and MTS, ROS takes place entirely in Erlenmeyer flasks. Samples are contained within the flasks with rumen fluid and prepared solutions; heated water baths and agitation are used to mimic the environment of the digestive tract.

Liebe et al. (2018) compared estimates of dRUP obtained using published results of studies that used either the MOB and MTS assay and observed that MOB predicted dRUP 6.2 percentage points greater than MTS. Ross (2013) compared MTS to ROS using a variety of feedstuffs including blood meal, soy products, and corn products, and observed that rumen protein degradability was 18 percentage points greater with the MTS assay but that total-tract protein digestibility was similar. To our knowledge, no research has yet been conducted to compare all 3 assays. Therefore, the objectives of this study were to determine CP rumen degradability and intestinal digestibility of HFM containing differing amounts of blood according to the MOB, MTS, and ROS assays. We hypothesized that the MOB and MTS assays would be more similar in their estimates than the ROS assay. Given that blood meal can but does not always (NRC, 2001) have a greater rumen degradability than HFM, we also hypothesized that HFM with more blood and HFM with less blood would differ in intestinal digestibility of CP.

Feedstuffs evaluated in this experiment differed in source of origin and by the amount of blood included in the feed. The feedstuffs were hydrolyzed feather meal low in blood (**FM**; from American Proteins Inc., Cumming, GA; Pilgrim's Pride Corporation, Mt. Pleasant, TX; Pilgrim's, Greeley, CO; River Valley Animal Foods, Robards, KY; and Simmons Foods, Siloam Springs, AR) and hydrolyzed feather meal with more blood (**FMB**; from Darling Ingredients Inc., Irving, TX; Mountaire Farms, Millsboro, DE; Pet Solutions, Danville, AR; River Valley Animal Foods, Sedalia, MO; and Sanimax, Green Bay, WI). One sample of hydrolyzed feather meal from each plant (5 FM and 5 FMB) were spot sampled, resulting in a total of 10 samples. The companies self-disclosed the nature of samples as containing blood or not but did not state the specific concentration of blood for those samples containing blood. Nonenzymatically browned soybean meal (SoyPass, LignoTech Florida LLC) was used as a standard for all methods. Before being subjected to the assays, feedstuffs were analyzed for DM (AOAC International, 2000), N (Leco FP-528 N Combustion Analyzer; Leco Corp.), neutral detergent insoluble crude protein (Leco FP-528 N Combustion Analyzer), acid detergent insoluble crude protein (Leco FP-528 N Combustion Analyzer), NDF (Van Soest et al., 1991), ADF (method 973.18; AOAC International, 2000), sugar (DuBois et al., 1956), ether extract (method 2003.05; AOAC International, 2006), ash (method 942.05; AOAC International, 2000), and minerals (method 985.01; AOAC International, 2000) by Cumberland Valley Analytical Services Inc. (Hagerstown, MD).

Before conducting the experiment, procedures using animals were approved by the University of Nebraska-Lincoln institutional animal care and use committee. Two multiparous Holstein cows (660 ± 33 kg of BW, 210 ± 17 DIM, 27.3 ± 8.00 kg/d of milk yield, 28.3 ± 2.92 kg/d of DMI) fitted with flexible ruminal and proximal duodenal cannulas were used for the MOB procedure. Cows were housed in tiestalls with continuous access to water and fed a late-lactation diet once daily at 1000 h. Ruminal degradations of CP were determined in situ and intestinal digestibilities were determined using the MOB technique as outlined by Paz et al. (2014). Paz et al. (2014) also showed that correction for microbial CP (**MCP**) contamination is more relevant in feedstuffs with a high NDF content. Because HFM technically contains no plant cell wall, no correction for MCP contamination was included.

Two dry, multiparous Jersey cows (482 ± 3 kg of BW, 89 ± 11 DIM, 33.7 ± 0.78 kg/d of milk yield, 21.3 ± 0.97 kg/d of DMI) fitted with flexible ruminal cannulas were used for the ruminal incubation portion of the MTS procedure. Cows were housed in tiestalls with continuous access to water and fed a dry cow diet once daily at 1000 h. Preparation, incubation, and washing of the nylon bags was completed following the same steps as described in the MOB assay (Paz et al., 2014). Following rumen incubation and washing, the remaining portions of the MTS assay were performed as described in Gargallo et al. (2006). Residues from the MOB and MTS assays were further analyzed for DM and N. Again, no correction for MCP was included.

All steps of the ROS assay were performed at Milk Specialties Global LLC (Eden Prairie, MN). Rumen fluid used to quantify rumen degradation was collected from 2 rumen-cannulated multiparous, lactating Holstein cows (647 ± 16.1 kg of BW, 163 ± 112 DIM, 40.7 ± 9.96 kg/d of milk yield, 23.2 ± 0.64 kg/d of DMI) housed offsite in a tiestall barn with continuous access to water and a lactating cow TMR. Samples were prepared and subjected to the ROS assay according to Ross et al. (2013). Upon completion, filters were dried at 105°C for 24 h in a drying oven (Hotpack Corp.) and then analyzed for DM and N (Leco FP-528 N Combustion Analyzer) by Cumberland Valley Analytical Services Inc.

The remaining composite residues from each assay were also analyzed by Cumberland Valley Analytical Services Inc. for DM (AOAC International, 2000) and N (Leco FP-528 N Combustion Analyzer). An inadequate amount of residue was available following analysis, so we could not determine and test the digestibility of AA. Each source of HFM was evaluated twice using each assay. In the case of the MOB and MTS assays, this replication was conducted by using 2 different cows. Data were analyzed using the PROC GLIMMIX function of SAS (9.4; SAS Institute Inc.). The model included the fixed effects of presence of blood and assay type as well as the interaction of presence of blood and assay type. All data are presented as least squares means ± largest standard error. The DIFF option was used for means comparisons between assay types. Significance was declared with a *P*-value ≤0.05.

Although the chemical composition of these 2 types of commercially available HFM were similar, small differences were observed (Table 1). Specifically, samples of FMB contained a higher concentration of DM, ash, and CP compared with FM samples. Additionally, crude fat was lower in FMB samples than in FM samples. Although Table 1 lists the NDF and ADF content of these feeds, HFM and blood meal do not contain any fiber, and these values are estimates of the portion of these feeds that are not broken down by neutral and acid detergent solutions. Differences in chemical composition between FM and FMB are likely due to the presence or absence of blood. According to NRC (2001), HFM has an ash content of 3.50%, a CP content of 92%, and a crude fat content of 4.6%, whereas blood meal has ash, CP, and crude fat contents of 2.50, 95.5, and 1.20%, respectively. The presence of blood in FMB results in a higher ash content because of the high iron content (2,453 mg/kg; NRC, 2001), and it likely dilutes the crude fat content because of the low crude fat content of blood. Differences may also be due to the species of animal processed in the facility and other differences in processing methods across plants (Cotanch et al., 2007). Variations in hydrolyzation and drying methods can also alter the chemical composition of the final product (Meeker and Hamilton, 2006), but the effect of these methods on chemical composition and on degradability and digestibility are beyond the scope of the current study.

**Table 1:** Chemical composition of hydrolyzed feather meal with and without blood (% of DM unless otherwise stated)

Item	Treatment^1^			
	FM		FMB	
	Mean	SD	Mean	SD
DM, % as-is	91.9	0.48	93.3	1.54
CP	90.5	2.14	91.9	1.94
NDF^2^	23.6	1.51	28.8	6.06
ADF^2^	3.23	1.13	2.38	1.54
ADICP^2^	4.96	1.02	4.24	1.07
NDICP^2^	21.7	2.84	27.0	5.15
Crude fat	8.58	1.51	7.08	1.73
Ash	2.74	1.13	6.19	3.24

1 FM = feather meal without added blood, FMB = feather meal with added blood. n = 5 per treatment.

2 Analyte is, by definition, a component of the plant cell wall, thus corresponding estimate is an artifact of the assay. ADICP = acid detergent insoluble CP; NDICP = neutral detergent insoluble CP.

In this study, digestibility of HFM was estimated using 3 assays: the MOB, MTS, and ROS assays. We were unable to conduct all assays simultaneously. Consequently, not all variance is strictly analytical but may include additional error associated with animal or site. The animals used were at different stages of lactation and fed different diets, which can affect the rumen degradation of protein (Broderick et al., 2004; Schadt et al., 2014). No interaction (*P* ≥ 0.397) was observed between type of HFM and assay; thus for clarity, the least squares means of these factors are reported in Table 2, Table 3. The estimates of digestibility of FM and FMB, according to the 3 assays, are listed in Table 2. A significant difference (*P* < 0.001) between assay type was observed in RUP. Although the mean RUP of MOB and MTS were similar, the mean RUP observed from the ROS assay was lowest. Although all samples were subjected to either a 16-h rumen incubation or 16-h incubation in rumen fluid, differences in the nature of the incubation may explain, at least in part, the observed differences in RUP. Specifically, samples for both MOB and MTS were incubated in nylon bags in situ, whereas the ROS incubation occurred with samples placed in a flask containing a mixture of Van Soest rumen buffer and rumen fluid under continuous CO_2_. In the case of the MOB and MTS assays, it is possible that some portion of soluble components contained in the samples could escape the bags but not necessarily be degraded or digested; this would lead to an overestimation of rumen degradability (Ross, 2013). On the other hand, incubation in flasks used by ROS does not allow for the removal of any products of microbial degradation, which could have a negative effect on fermentation (Coleman, 1985). Additionally, the presence of fat could have limited microbial activity by having an antimicrobial effect or creating a barrier between feed particles and microbes (Jenkins, 1993). Should this occur, a decrease in microbial activity would lead to less digestion and thus a lower RUP, which was observed in ROS. However, this often occurs in the rumen under normal conditions and in other assays, so the accumulation of products from microbial degradation is likely the cause of a lower RUP. Interestingly, RUP was lowest in the ROS assay, suggesting, at least in the case of HFM, that degradation was occurring, perhaps even to a greater extent than that occurring in situ. It should be noted that the ROS assay was not originally designed to estimate RUP per se but rather to isolate RUP residue that would reach the small intestine in vivo to estimate apparent total-tract CP digestibility (**TTCPd**). Additional research should be conducted to evaluate factors that affect RUP concentration using ROS to ensure that the residue isolated is similar to RUP in vivo.

**Table 2:** Rumen and intestinal digestion of protein (% of DM, unless otherwise noted) of hydrolyzed feather meal with and without blood for the mobile bag (MOB), modified three-step (MTS), and Ross (ROS) assays

Item	Assay^1^			SEM	*P*-value
	MOB^2^	MTS^3^	ROS^4^		
RUP, % of CP	77.8^a^	71.9^a^	41.9^b^	1.80	<0.001
TTCPd,^5^ % of DM	69.4^b^	70.0^b^	94.0^a^	2.67	<0.001
dRUP,^6^ % of RUP	61.1^a^	58.1^a^	62.2^a^	3.53	0.697
RUP digested, % of DM	42.9^a^	38.0^a^	23.4^b^	2.28	<0.001

a,b Means with different superscripts in the same row differ (*P* ≤ 0.05).

1 n = 5.

2 MOB = nonenzymatically browned soybean meal control (n = 2); RUP = 85.5 ± 5.72, TTCPd = 83.7 ± 10.8, dRUP = 76.03 ± 10.8, RUP digested = 67.9 ± 0.31.

3 MTS = nonenzymatically browned soybean meal control (n = 4); RUP = 81.1 ± 6.01, TTCPd = 95.6 ± 2.13, dRUP = 94.0 ± 2.88, RUP digested = 76.2 ± 3.30.

4 ROS = nonenzymatically browned soybean meal control (n = 2); RUP = 28.0 ± 0.55, TTCPd = 98.0 ± 0.66, dRUP = 92.9 ± 2.34, RUP digested = 26.0 ± 0.15.

5 TTCPd = apparent total-tract CP digestibility.

6 dRUP = intestinal digestibility of RUP.

**Table 3:** Rumen and intestinal digestion of protein (% of DM, unless otherwise noted) of hydrolyzed feather meal with (FMB) and without blood (FM)

Item	Feedstuff^1^, ^2^		SEM	*P*-value
	FM	FMB		
RUP, % of CP	60.8	66.9	1.47	0.007
TTCPd,^3^ % of DM	75.4	73.5	2.18	0.531
dRUP,^4^ % of RUP	60.1	60.8	2.88	0.859
RUP digested, % of DM	32.6	37.0	1.86	0.114

1 n = 5.

2 Control = nonenzymatically browned soybean meal (n = 8); RUP = 68.9 ± 25.6, TTCPd = 91.3 ± 10.5, dRUP = 90.1 ± 7.22, RUP digested = 61.5 ± 22.4.

3 TTCPd = total-tract CP digestibility.

4 dRUP = intestinal digestibility of RUP.

Despite differences in RUP, the assay type did not affect estimates of dRUP (*P* = 0.697). Both the MTS and ROS assays were developed to provide a rapid, more affordable, and less labor-intensive alternative to the MOB assay. To simulate intestinal digestion, both assays used steady agitation and a solution of various buffers and enzymes. The MTS assay still relies upon rumen incubation of samples but simulates intestinal digestion with a buffer-enzyme solution (Gargallo et al., 2006), whereas the ROS assay incubates samples in individual Erlenmeyer flasks (Ross, 2013). The similarity among the dRUP values of these assays provides preliminary evidence that all 3 may be viable options to estimate the intestinal digestibility of animal-based protein feedstuffs. It should be noted that analytical differences existed in procedural steps isolating RUP residue between the ROS assay and the MOB and MTS assays. As a consequence, the nature of residue used in estimating intestinal digestibility likely also differed in nature. This is important because it is well understood that intestinal digestibility of RUP is not a constant value but is inversely related to ruminal digestion and, in vivo, is affected by the rumen environment and outflow of digesta (Hvelplund et al., 1992). Furthermore, with the observed similarity in dRUP, the amount of RUP digested differed significantly (*P* < 0.001) across assays. The MOB and MTS assays yielded similar amounts, whereas ROS resulted in a smaller amount of RUP digested. This response was likely due to the difference between rumen incubation and incubation in rumen fluid. The difference in digested RUP can have significant effects on outputs in ration formulation when it comes to determining sources of bypass protein as well as the most affordable feedstuff per unit of protein supplied. Although the MOB and MTS values were similar for TTCPd, the ROS values were observed to be higher. The difference observed among these assays is likely a residual effect of the differences observed in rumen digestibility.

Table 3 lists the mean digestibility estimates of FM and FMB. A significant response (*P* = 0.007) was observed for RUP expressed as a proportion of CP. Feather meal and feather meal with some viscera both have the same RUP content as listed in the NRC (2001). The exact reason for the differences in RUP observed is unknown, but like the differences observed in chemical composition, the presence of blood and processing methods may affect the digestibility of the sample. Blood meal has a higher RUP content (77.5%, forage 50% of DMI) than HFM (65.4%, forage 50% of DMI), so the addition of blood would increase the RUP content of the product (NRC, 2001). The processing methods of each of the samples used in the present study are unknown. Thus, it is unclear what differences in processing could cause the difference in RUP between FM and FMB. Because we only replicated FM and FMB and did not replicate within a plant, we could not test for plant effects.

No differences (*P* = 0.859) in dRUP were observed between FM and FMB (average of 60.5 ± 0.49%). Our mean estimate of dRUP of HFM is approximately 10 percentage points lower than that listed by NRC (2001) for feather meal with some viscera (70%) but is similar to that reported for HFM (65%). The method by which blood meal is processed likely affects digestibility. This is supported by a study conducted in rats showing that intestinal protein digestibility of blood meal varied from 17.0 to 94.6%, and this difference was speculated to be in response to differences in drying methods (Moughan et al., 1999). Despite differences in rumen protein digestibility, the presence of blood had no effect (*P* = 0.531) on TTCPd. This is contrary to our expectation, which was that FMB would have a higher digestibility than FM. Waltz et al. (1989) evaluated the total-tract digestibility of poultry blood meal, feather meal, and an equal blend of these feeds using MOB. In that study, blood meal was observed to have the greatest digestibility (43%) followed by the blend (28%) and feather meal (21%). In the present study, the concentration of blood included in the batches represented by our samples is unknown, but a varying inclusion level of blood could explain why RUP, but not dRUP, increased. However, because of the increased RUP content of FMB, the amount of RUP digested was numerically higher (*P* = 0.114) for FMB than for FM.

The aim of this study was to compare 3 assays used to determine protein digestibility of HFM in ruminants. Although assays differed in the extent to which incubations were done in situ, estimation of intestinal digestion of bypass protein was similar across assays. However, due to differences in estimates of RUP, the amount of RUP digested was quite different across assays. More research is needed to compare and further validate these assays simultaneously with a variety of feedstuffs. Furthermore, despite research suggesting that the inclusion of blood alters the chemical composition and digestibility of HFM, our results suggest that differences exist in RUP content but not in estimates of dRUP. This implies that intestinal digestibility is not affected by the inclusion of blood during the rendering process, but the amount of RUP digested is numerically increased with blood inclusion.
